# Digital Phenotyping of Emotion Dysregulation Across Lifespan Transitions to Better Understand Psychopathology Risk

**DOI:** 10.3389/fpsyt.2021.618442

**Published:** 2021-05-24

**Authors:** Robert D. Vlisides-Henry, Mengyu Gao, Leah Thomas, Parisa R. Kaliush, Elisabeth Conradt, Sheila E. Crowell

**Affiliations:** ^1^Department of Psychology, University of Utah, Salt Lake City, UT, United States; ^2^Department of Obstetrics and Gynecology, University of Utah, Salt Lake City, UT, United States; ^3^Department of Pediatrics, University of Utah, Salt Lake City, UT, United States; ^4^Department of Psychiatry, University of Utah, Salt Lake City, UT, United States

**Keywords:** digital phenotyping, emotion dysregulation, perinatal, emerging adulthood, adolescence, psychopathology risk, lifespan transitions, passive monitoring

## Abstract

Ethical and consensual digital phenotyping through smartphone activity (i. e., passive behavior monitoring) permits measurement of temporal risk trajectories unlike ever before. This data collection modality may be particularly well-suited for capturing emotion dysregulation, a transdiagnostic risk factor for psychopathology, across lifespan transitions. Adolescence, emerging adulthood, and perinatal transitions are particularly sensitive developmental periods, often marked by increased distress. These participant groups are typically assessed with laboratory-based methods that can be costly and burdensome. Passive monitoring presents a relatively cost-effective and unobtrusive way to gather rich and objective information about emotion dysregulation and risk behaviors. We first discuss key theoretically-driven concepts pertaining to emotion dysregulation and passive monitoring. We then identify variables that can be measured passively and hold promise for better understanding emotion dysregulation. For example, two strong markers of emotion dysregulation are sleep disturbance and problematic use of Internet/social media (i.e., use that prompts negative emotions/outcomes). Variables related to mobility are also potentially useful markers, though these variables should be tailored to fit unique features of each developmental stage. Finally, we offer our perspective on candidate digital variables that may prove useful for each developmental transition. Smartphone-based passive monitoring is a rigorous method that can elucidate psychopathology risk across human development. Nonetheless, its use requires researchers to weigh unique ethical considerations, examine relevant theory, and consider developmentally-specific lifespan features that may affect implementation.

## Introduction

In our increasingly digital society, a person's unique technological interactions can provide meaningful information about their mental health symptoms (1). One device with great potential for revealing individual-level behavior is the smartphone. Digital phenotyping, limited here to smartphone-based passive monitoring, is defined as using smartphone data to better understand human traits, behaviors, and functioning. Smartphone-based assessment of daily activity provides a novel window into multifaceted aspects of behavior, which may in turn help mental health researchers better understand risk trajectories (2–5).

Passive monitoring thus allows for highly objective and dynamic assessment of psychopathology-related behavior that extends beyond the confines of diagnostic syndromes. Emotion dysregulation underlies risk for diverse forms of psychopathology across the lifespan (6), and may be assessed well with passive monitoring approaches. We argue that passive monitoring holds particular benefit for the assessment of emotion dysregulation across major lifespan transitions. The primary goal of this paper is to identify passive measures that may prove fruitful for understanding daily life emotion dysregulation. We also discuss future directions, limitations, and highlight persistent challenges in this area, including navigating participant privacy and data security.

## Key Terms: Passive Monitoring, Emotion Dysregulation, and Lifespan Transitions

**Figure 1:**
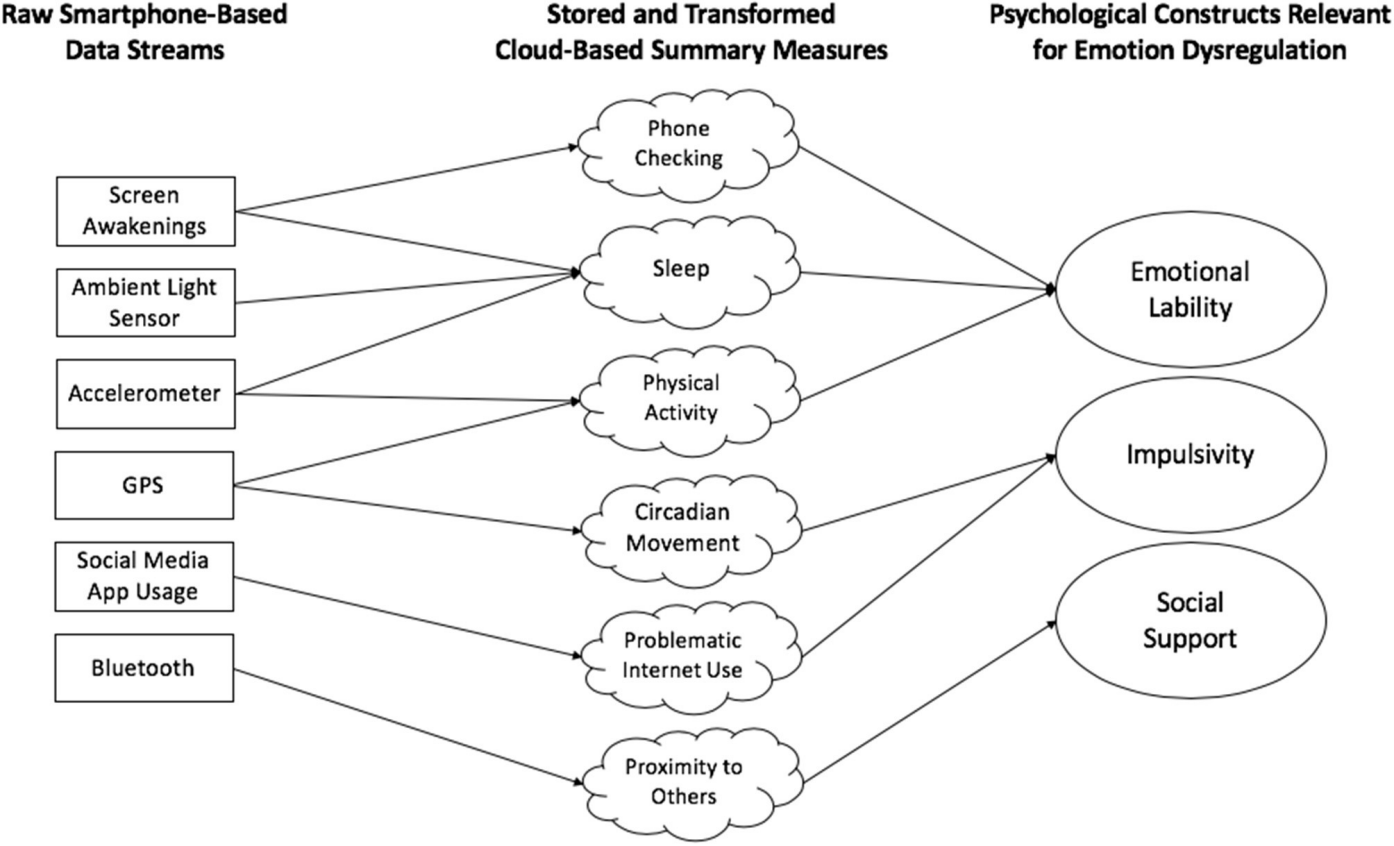
Conceptual illustration of current smartphone-based passive monitoring methods. Raw data streams are collected directly from smartphones and then converted into more useful variables/measures through cloud-based interfaces. These measures convey meaning about relevant constructs—in this case, emotion dysregulation. Note that, for simplicity, this figure conveys merely a small sample of current possible data streams and variables, and many others exist.

In this paper, we restrict our discussion of digital phenotyping to consensual and ethical passive monitoring of behavior *in situ* (1, 3). Passive monitoring refers to collection of raw smartphone-based data streams, requiring little participant effort. Smartphones are regularly improved and modified, so it is important to situate this discussion in the context of existing technology. In this manuscript, we do not discuss digital data collection that occurs without participant awareness, such as web scraping or anonymized social media analysis. Smartphone-based passive monitoring is a structured, consented process, in which participants are aware that researchers will gather various forms of smartphone data to understand their behavior. Current examples of smartphone-based raw data streams include screen activation time, location via GPS, Bluetooth activity, and texting speed, among others. Through passive monitoring, raw data streams are manipulated into more meaningful variables. For instance, by gathering ambient light and accelerometry data, researchers can currently estimate sleep time, and with GPS, one can calculate various features of mobility ((7, 8); Figure 1). When paired with self-reported symptom measures, passive monitoring can provide more comprehensive mental health data, such as onset of psychotic and manic episodes (9–13). Given its multifaceted capabilities, passive monitoring can help reveal discrete mental health states. Passive monitoring methods may also lead to improved understanding and prediction of risk factors for psychopathology that cut across a number of disorders, such as emotion dysregulation.

Emotion dysregulation refers to emotional experiences and expressions that are over- and/or under-controlled in a manner that interferes with goal-related behavior (14). Emotion dysregulation is a complex multilevel construct, with trait- and state-level components, associated with numerous psychiatric disorders (15, 16). This construct also predisposes individuals across the lifespan to risk for maladaptive coping responses to distress (e.g., self-harm, substance use; (17–19)). For example, children, adolescents, and young adults with emotion regulation difficulties are at heightened risk for internalizing and externalizing syndromes as they develop (14, 20, 21). Dysregulated emotion is a function of both trait-level individual differences in impulsivity and anxiety, as well as state-level fluctuations in affect (15). Passive monitoring methods are particularly well-positioned to tap into state dynamics but their ability to do so is no doubt a function of person- and observation-level sampling. With a large number of participants, individual differences will emerge more readily, and observations will be a function of between-person effects. Passive methods lend themselves well to the gathering of numerous within-person observations, leading to a stronger understanding of within-person dynamics (1, 4). Regardless, the multifaceted and broad nature of emotion dysregulation make it an ideal construct for digital methods, particularly for individuals navigating major lifespan transitions, who must manage significant daily stress and adjustment.

Lifespan transitions often confer risk. For instance, rates of internalizing and externalizing psychopathology increase in adolescence in part due to hormonal shifts and changes in social groups (22, 23). Emerging adults also have relative increases in risk due to continued neurological development, shifts in autonomy, and identity formation (24, 25). Additionally, the perinatal period is a critical transition, leading to increased psychiatric sensitivity and profound changes in identity (e.g., labeling oneself as a “parent”), the effects of which can confer risk for longstanding neurodevelopmental outcomes in infants (26, 27). Transitions also involve a great deal of adjustment. Adolescents navigate high school, young adults often move out and seek independent careers, and perinatal women and their partners manage newfound health challenges and parenting stress—all of which make it difficult to gather rich psychopathology data in the laboratory. Smartphone-based monitoring of behavior represents an innovative way to understand dynamic, daily life, and transdiagnostic risk trajectories.

## Lifespan Transitions and Passive Monitoring of Emotion Dysregulation

**Figure 2:**
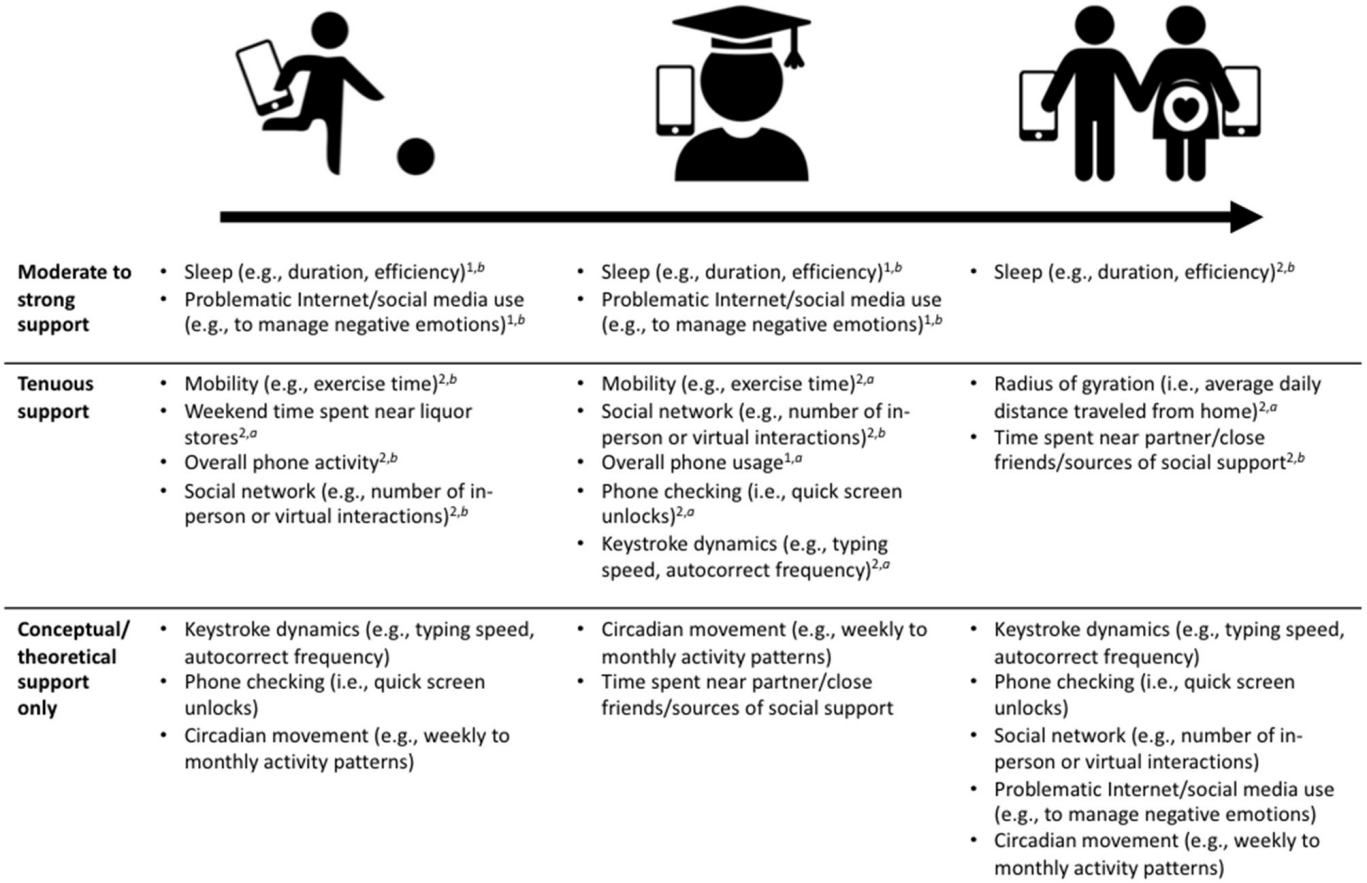
Use of digital phenotyping summary measures as markers of emotion dysregulation across major lifespan transitions. Moderate to strong empirical support: >2 peer-reviewed studies. Tenuous empirical support: 1–2 peer-reviewed studies. Conceptual/theoretical support only: author perspective, important measures for future study. ^1^Use of this variable has been supported by at least one study demonstrating its association with a “gold standard” emotion regulation measure (e.g., affective lability, difficulty controlling emotions). ^2^Use of this variable is supported by evidence of its association with indirect markers of emotion dysregulation (e.g., depression, anxiety, well-being). ^a^Cited study (or studies) gathered evidence through smartphone-based passive monitoring. ^b^Evidence acquired without passive monitoring. Note: no notation used for the last row because these items are not currently backed by evidence, representing the authors' perspective only.

In this manuscript, we explore three lifespan transitions marked by both smartphone use and increased psychopathology risk: adolescence, emerging adulthood, and the perinatal period. We discuss key features of each transition with respect to emotion dysregulation as well as how passive monitoring might be applied. We chose to limit our discussion to these three transitions due to space constraints, and because smartphone ownership is limited in childhood. In addition, we outline potential passive measures of emotion dysregulation by strength of support in the literature (moderate-strong, tenuous, or conceptual/author perspective). We also note the extent to which each highlighted measure has been associated with emotion dysregulation directly (e.g., with a “gold standard” measure) or indirectly (e.g., associated with an emotion regulation disorder, such as depression), and if the measure has been gathered via smartphone-based passive means in that population (see Figure 2).

### Adolescence Transition

Adolescence comprises the time between late childhood and emerging adulthood (28). Specific age range definitions vary but we restrict our definition to the 12–17 age range, avoiding overlap with childhood and emerging adulthood and capturing pubertal onset for most people. Though there is much literature on media exposure in childhood (especially violent media) in relation to psychopathology risk (29), this research is beyond the scope of the current paper. Very little smartphone-based passive monitoring research has been conducted among children (as few children under 12 currently own smartphones). Moreover, a discussion of ethics with respect to smartphone monitoring in childhood warrants a more in-depth analysis than can be done here.

Post-pubertal adolescent brains undergo extraordinary changes in structure and function, including gray matter pruning and increased connectivity in the prefrontal cortex (30). By the end of puberty, adolescents have experienced significant maturation of the hypothalamic-pituitary-adrenal (HPA) axis and respective sexual organs (31), as well as overall decreases in serotonin and increases in dopamine (32, 33). Complex interactions between hormones, brain maturation and functioning, and genomic (de)activation increase adolescent risk for mental health problems (28, 33, 34). Given the risk and stress of this transition, it follows that an approach that minimizes research participation burden, such as daily life smartphone monitoring, could be fruitful.

Two variables that can be passively monitored appear to have at least moderately strong support as emotion dysregulation indices in this stage: sleep disturbance and problematic Internet usage. Countless studies have demonstrated that poor sleep, often measured via self-report or actigraphy, is a viable index of mood disorder risk across the lifespan (34–38). Smartphone monitoring (i.e., raw data from ambient light sensor and accelerometer) has been used to assess sleep across populations, often validated by actigraphy (39–43). In one study, researchers demonstrated a clear link between emotion regulation problems and poor adolescent sleep (36). Additionally, problematic Internet/social media use, or technology use as a means of regulating distress (e.g., using a smartphone to distract oneself from anxiousness), has also been linked to psychopathology, and may be explained by emotion dysregulation across diverse samples (20, 44–48), though in none of these studies was passive monitoring used. Nevertheless, adolescence is a time of increased emotional responsivity (due to increases in limbic volume) with incomplete prefrontal cortex development, which may explain adolescents' propensity to unhealthy emotion regulation strategies (28). Although empirical support has been acquired without passive monitoring, abundant evidence suggests a link between problematic Internet use, sleep, and emotion dysregulation, indicating at least moderate support for these indices.

There are several passive measures with weak or tenuous support. Many of these measures still require rigorous validation in the adolescent context. Physical activity is related to general well-being in adolescents [though untested with passive monitoring methods; (49, 50)]. Robust social networks and close relationships also associate with less adolescent emotion dysregulation (51, 52). Network analyses have revealed interesting contagion effects across adolescent peer groups (53), which could potentially be captured through passive monitoring methods. Contagion-like effects could be assessed through social media app activity and through the use of phone-phone closeness via Bluetooth. Finally, passive monitoring studies have shown that adolescent weekend proximity to liquor stores (54) and daily smartphone use (44) may index behavior problems and suicidality, respectively—both proxies for dysregulated emotion.

We have identified several untested candidate measures that may prove useful for detecting emotion dysregulation. Keystroke dynamics (i.e., texting speed, autocorrect frequency) and frequent, brief phone checks can detect emotion regulation problems and may be worth examining with adolescents (13, 44). Both may be useful because individuals who are highly dysregulated struggle to allocate attention effectively (14, 15), similar to problematic Internet/social media use. Finally, circadian movement, a measure of variability in GPS locations over a day or week, may also index dysregulation. Individuals with more irregular daily patterns may have more financial or social insecurity and could be more dysregulated, making this a fascinating candidate marker for future research.

### Emerging Adulthood Transition

We define emerging adulthood broadly as between age 18 and the late 20s, as this period captures the time between adolescence and the tapering of neurological development (55). Like adolescence, emerging adulthood is a time of increases in psychopathology risk, relative to childhood and adulthood. This change in risk for mental illness may occur because many emerging adults experience exponential growth in autonomy and identity formation, often pursuing higher education, careers, and intimate relationships (56). Yet there is also more lifestyle and day-to-day heterogeneity in this period relative to adolescence. Adolescents typically attend school, making it relatively simple for researchers to apply digital methods uniformly. However, much of what is known about emerging adults is limited to college students. Thus, passive monitoring may provide an effective way to better understand heterogeneous mental health risk trajectories in this population.

Similar to adolescence, effective passive markers for emotion dysregulation include both sleep and problematic Internet/social media use, the latter of which is often defined as Internet/social media use that leads to social, emotional, or behavioral problems or negative outcomes (56). For example, using the Internet as a primary means for managing distress or avoidance might be considered problematic, especially because prefrontal cortices only finish developing by the end of young adulthood (57). Many non-digital studies have demonstrated links between self-reported sleep and emotion dysregulation in college student samples (58, 59). Note that there are several studies on passively-monitored sleep in adults, not young adults (35). Likewise, self-report studies of college students link problematic Internet/social media use and dysregulated emotion (60, 61). There is a need for more smartphone-based assessment of these measures, though the large number of significant self-report studies suggests they are at least moderately supported indices.

There are also several passive measures that have at least weak support as emotion dysregulation indices. Some have been examined in only adult populations, and others have been examined via self-report in a limited fashion. Similar to adolescents, there is good reason to suspect that daily mobility and interpersonal interaction patterns, measured, for instance, via GPS and Bluetooth, might index emotion dysregulation. As adolescents transition into young adulthood, frontal lobes tend to become increasingly active, leading to increased desire for social engagement and interpersonal risk taking (28, 55). Among adults in general, smartphone-derived mobility indices, including number of locations visited, normalized entropy (i.e., variability in time spent at significant locations), and time spent at home predicted depression and anxiety, though associations with emotion dysregulation itself are untested (62–64). Additionally, young adults who engage in effective interpersonal emotion regulation tend to have greater popularity and social network size, indexed via peer report and Twitter following [though not passive monitoring; (65)]. This aligns with numerous emotion regulation theories, such that affect is regulated through both intrapersonal and interpersonal means (66, 67).

Additionally, there is evidence that total daily phone usage may predict depression in adults (62, 68). However, a more nuanced usage measure is phone checking, a common behavior among young adults (69). Frequent checking, defined here as brief lapses between screen lock-unlock, may index ineffective regulation of distress and reassurance seeking, making it a potential measure of utility (45, 70, 71). Finally, keystroke dynamics (e.g., typing more quickly during a manic episode) have been examined in adult clinical populations and are worth examining in emerging adults, given that this measure predicts mood states (13).

Two additional measures that may warrant investigation are circadian movement (see “Adolescence”) and time spent near a close partner. This latter marker could be approximated through Bluetooth. When activated, smartphones can use Bluetooth to detect proximity to other smartphones. Although the identity of non-participant smartphones would be withheld, researchers could determine if the same smartphone is in proximity repeatedly, as one would expect with a close friend or partner (8). Oftentimes, healthy relationships with close friends and romantic partners associate with better regulated emotion (72), so it stands to reason that this could be true when examined via passive monitoring.

### Perinatal Transition

The perinatal period is a time of significant change for women and families. In less than a year, pregnant women experience numerous profound neurobiological changes, matched only by fetal and pubertal development, likely to facilitate child bearing and parent-infant bonding (27). Preparing for and raising a new child can cause significant financial and social stress, as pregnant women must navigate newfound daily routines, finances, health concerns, and identity shifts (73). Thus, this lifespan transition confers increased mental health risk, yet much perinatal psychopathology is under- or misdiagnosed (74). Perinatal mental health problems can manifest in highly idiosyncratic ways, unique from other parts of the lifespan (e.g., perinatal depression is more likely to have anxious and obsessive-compulsive features), and symptoms are also quite variable from day to day (75, 76). Moreover, perinatal mental health affects not only a pregnant woman's long-term health, but that of her newborn. Significant stress, depression, and emotion dysregulation during pregnancy may predispose newborns to maladaptive development (27, 77, 78). These intergenerational implications underscore the need to better understand risk during this stage.

To our knowledge, little passive monitoring research has been done with perinatal women and none with their partners. Likely, the most supported emotion dysregulation measure that can be assessed with passive monitoring is sleep disturbance. Though mild to moderate sleep disturbance is quite common throughout this transition, not all perinatal women develop sleep disorders (79). Though empirical work is lacking, non-digital studies have shown that prenatal and postnatal sleep disturbances are associated with postpartum depression and other indirect markers of emotion regulation problems, suggesting this could be a useful index (80–82).

One passive measure with at least tenuous promise is “radius of gyration,” or the average daily distance traveled from home (8). In one of the only published studies that used passive monitoring during pregnancy, researchers found that a lower radius of gyration associated with day-to-day change scores in self-reported mood, though not daily mood itself (83). In this same study, smartphone-detected mobility (i.e., daily distance traveled on foot) did *not* predict self-reported emotion. This finding may speak to the unique features of this transition. Pregnant women tend to move less over the course of gestation, meaning that mobility may not be as consistently linked to emotion as it is for adolescents and emerging adults. Also, because smartphones can be used to detect proximity to other individuals, the presence of supportive attachment figures during the perinatal period seems to help reduce distress and, indirectly, lead to more effective emotion regulation (84). Indeed, prenatal maternal brains experience a remarkable amount of “rewiring” in the prefrontal cortex, likely to facilitate attachment bonds with partners and with their new child (27). Assessing time spent in proximity with others via Bluetooth could help researchers understand regulatory problems in pregnancy.

Due to a lack of research in this area, there are many additional passive measures that, from our perspective, may enrich our understanding of perinatal emotion dysregulation. These proposed measures have already been discussed in detail throughout this paper. For instance, keystroke dynamics may prove a useful measure of mood dysregulation during the perinatal period; individuals who are dysregulated may have high variability in texting speed, autocorrect frequency, etc. across temporal shifts in negative affect (13). We also propose phone checking, social network assessment (both in-person and virtual), and problematic Internet/social media use as candidate emotion dysregulation indices, as they appear effective at other points across the lifespan. Last, given the theoretical justification described previously, researchers may also consider validating circadian movement as an emotion dysregulation index during the perinatal transition.

## Discussion

Emotion dysregulation is a heterogeneous, transdiagnostic risk factor with health implications across the lifespan. Smartphone-based passive monitoring presents researchers and clinicians with a relatively inexpensive way to monitor psychopathology-related behavior. However, much passive monitoring research has relied on exploratory and atheoretical methods, ultimately inhibiting advancement (43). We presented key concepts and articulated a framework that will be helpful for understanding how passive monitoring might be used for daily life emotion dysregulation assessment across three major lifespan transitions: adolescence, emerging adulthood, and the perinatal transition. The most robust indices are likely smartphone-based assessment of sleep disturbance and problematic Internet/social media use, followed by derivations of mobility, sociality, and phone activity. Given that most research cited consists of self-report and other types of measurement, there is a critical need to validate all of these indices with passive, digital methods. As recommended (85), we encourage digital researchers to simultaneously gather daily life self-reports (i.e., ecological assessment).

There are several limitations and caveats to note as well. The passive monitoring markers outlined likely have complex, bidirectional relations with emotion dysregulation. For instance, numerous researchers have debated the extent to which sleep issues predispose one to regulatory problems or if sleep problems are a consequence of emotion dysregulation (86). For the purposes of this paper, underlying mechanisms are not crucial; our focus was on the utility of a potential marker. To estimate causality, researchers must use intensive longitudinal approaches with well-validated, multilevel measurement (87, 88). We expect these designs will be even easier with passive monitoring. Additionally, as described earlier, it is difficult to disentangle the extent to which these measures index state- or trait-level emotion dysregulation. Specific study designs will be helpful in doing so.

Additionally, the indices we discussed are not exhaustive. We have scratched the surface on how passive monitoring can inform psychopathology. Emerging research is highlighting how diverse technological interactions can help create comprehensive digital phenotypes of an individual's mental health. Cutting-edge studies show how to integrate multiple aspects of smartphone monitoring, wearable physiological technology, and built-in machine learning algorithms (2, 89). This type of work has incredible potential for increasing scalability and translation of mental health findings. For instance, one potential intervention involves using smart home features to lock gun safes and alert clinicians when individuals are detected to be in high distress [e.g., through voice sampling and texting; (90)].

However, exciting this type of work could be, researchers must remain attentive to ethical issues at play. Passive monitoring scientists balance participant privacy and data security with a need for rich data. Researchers should follow recommended data protection strategies from passive monitoring experts; for instance, it is important for scientists to store their data in encrypted servers, used hashed phone/Wifi identifiers (to avoid leaving identifying cellphone information in a dataset), and only publicly share de-identified data (1, 6). For instance, one passive monitoring platform known as Beiwe uses front-end and back-end interfaces, and identifiable participant data remain in the front-end server (8).

Ethics becomes especially complicated when working with adolescents, as navigating parent-child dynamics and dyads in the laboratory already has its own challenges (91), let alone outside of the laboratory. Researchers should take several additional steps to protect human rights. Due to the ongoing nature of smartphone-based data collection, it is important for researchers to provide not only detailed informed consent at study onset but opportunities for participants to re-consent and opt out if desired. Additionally, as mentioned, due to the sheer volume of highly sensitive data, researchers should take advantage of encrypted cloud-based servers that de-identify participant information prior to storage. Researchers should also consider explaining, in detail, how passive monitoring works during the informed consent process, and ensuring understanding when working with minors. It may be important to emphasize that only consented data are gathered, meaning data beyond what a smartphone can currently collect automatically are unobtainable (typically specific app usage, texting content, etc.). Researchers should do as much as they can to reassure participants that they will not be “watched” in real time; passive monitoring data go through numerous cloud-based data checks prior to becoming visible to researchers (8; Figure 1). Although a detailed discussion of these issues is beyond the scope of this manuscript, several excellent resources exist already (2, 87, 92, 93).

To advance the science of psychiatry, researchers have called for targeted application of digital methods to understand specific daily life behaviors underlying psychopathology risk and maintenance (9). Passive monitoring of emotion dysregulation is a complex and emerging area of interest (94). By understanding transdiagnostic risk factors across the lifespan through cutting-edge digital methods, we will enrich our understanding of psychopathology mechanisms and treatment directions. There is still much to be learned about how to advance this research area, and just as much critical knowledge to be gained.

## Data Availability Statement

The original contributions presented in the study are included in the article/supplementary material, further inquiries can be directed to the corresponding author/s.

## Author Contributions

RV-H conceptualized the structure and perspectives articulated in this manuscript and wrote the majority of the paper. MG, LT, PK, and EC provided editing, feedback, and comments on the writing and figures to strengthen the paper. SC provided detailed feedback and edits on the writing and contributed significantly to the conceptualization of the paper's themes. All authors are accountable for the accuracy and integrity of this work.

## Conflict of Interest

The authors declare that the research was conducted in the absence of any commercial or financial relationships that could be construed as a potential conflict of interest.

## References

[1] Onnela JP. Opportunities and challenges in the collection and analysis of digital phenotyping data. Neuropsychopharmacol. (2021) 46:45–54. 10.1038/s41386-020-0771-3 PMC7688649 32679583

[2] Montag C Sindermann C Baumeister H. Digital phenotyping in psychological and medical sciences: a reflection about necessary prerequisites to reduce harm and increase benefits. Curr Opin Psychol. (2020) 36:19–24. 10.1016/j.copsyc.2020.03.013 32361334

[3] Onnela J-P Rauch SL. Harnessing smartphone-based digital phenotyping to enhance behavioral and mental health. Neuropsychopharmacol. (2016) 41:1691–6. 10.1038/npp.2016.7 26818126 PMC4869063

[4] Torous J Onnela J-P Keshavan M. New dimensions and new tools to realize the potential of RDoC: digital phenotyping via smartphones and connected devices. Transl Psychiat. (2017) 7:e1053. 10.1038/tp.2017.25 28267146 PMC5416670

[5] Torous J Staples P Barnett I Sandoval LR Keshavan M Onnela J-P. Characterizing the clinical relevance of digital phenotyping data quality with applications to a cohort with schizophrenia. NPJ Digit. (2018) 1:1–9. 10.1038/s41746-018-0022-8 31304300 PMC6550248

[6] Beauchaine TP Crowell SE.(editors.). The Oxford Handbook of Emotion Dysregulation. New York, NY: Oxford University Press (2020).

[7] Sarda A Munuswamy S Sarda S Subramanian V. Using passive smartphone sensing for improved risk stratification of patients with depression and diabetes: cross-sectional observational study. JMIR mHealth uHealth. (2019) 7:e11041. 10.2196/11041 30694197 PMC6371066

[8] Onnela Lab. Beiwe wiki. Available online at: https://github.com/onnela-lab/beiwe/wiki (accessed September 15, 2020).

[9] Huckvale K Venkatesh S Christensen H. Toward clinical digital phenotyping: a timely opportunity to consider purpose, quality, and safety. NPJ Digit. (2019) 2:1–11. 10.1038/s41746-019-0166-1 31508498 PMC6731256

[10] Raballo A. Digital phenotyping: an overarching framework to capture our extended mental states. Lancet Psychiat. (2018) 5:194–5. 10.1016/S2215-0366(18)30054-3 29482758

[11] Insel TR. Digital phenotyping: technology for a new science of behavior. JAMA. (2017) 318:1215–6. 10.1001/jama.2017.11295 28973224

[12] Barnett I Torous J Staples P Sandoval L Keshavan M Onnela. Relapse prediction in schizophrenia through digital phenotyping: a pilot study. Neuropsychopharmacol. (2018) 43:1660–6. 10.1038/s41386-018-0030-z 29511333 PMC6006347

[13] Zulueta J Piscitello A Rasic M Easter R Babu P Langenecker SA. Predicting mood disturbance severity with mobile phone keystroke metadata: a biaffect digital phenotyping study. JMIR. (2018) 20:e241. 10.2196/jmir.9775 30030209 PMC6076371

[14] Beauchaine TP Zisner A. Motivation, emotion regulation, and the latent structure of psychopathology: an integrative and convergent historical perspective. Int J Psychophysiol. (2017) 119:108–18. 10.1016/j.ijpsycho.2016.12.014 28057475

[15] Beauchaine TP. Future directions in emotion dysregulation and youth psychopathology. J Clin Child Adolesc Psychol. (2015) 44:875–96. 10.1080/15374416.2015.1038827 26016727

[16] Crowell SE Vlisides-Henry RD Kaliush PR. Emotion generation, regulation, and dysregulation as multilevel transdiagnostic constructs. In: Beauchaine TP Crowell SE editors. The Oxford Handbook of Emotion Dysregulation. New York, NY: Oxford University Press (2020). p. 85–98. 10.1093/oxfordhb/9780190689285.013.7

[17] Crowell SE Price CJ Puzia ME Yaptangco M Cheng SC. Emotion dysregulation and autonomic responses to film, rumination, and body awareness: extending psychophysiological research to a naturalistic clinical setting and a chemically dependent female sample. Psychophysiology. (2017) 54:713–23. 10.1111/psyp.12838 28251663 PMC5522618

[18] Crowell SE Baucom BR Yaptangco M Bride D Hsiao R McCauley E.. Emotion dysregulation and dyadic conflict in depressed and typical adolescents: evaluating concordance across psychophysiological and observational measures. Biol Psychol. (2014) 98:50–8. 10.1016/j.biopsycho.2014.02.009 24607894 PMC4026166

[19] Crowell SE Beauchaine TP McCauley E Smith CJ Stevens AL Sylvers P. Psychological, autonomic, and serotonergic correlates of parasuicide among adolescent girls. Dev Psychopathol. (2005) 17:1105–27. 10.1017/S0954579405050522 16613433

[20] Kim J Cicchetti D. Longitudinal pathways linking child maltreatment, emotion regulation, peer relations, and psychopathology. J Clin Child Adolesc Psychol. (2010) 51:706–16. 10.1111/j.1469-7610.2009.02202.x 20050965 PMC3397665

[21] McLaughlin KA Hatzenbuehler ML Mennin DS Nolen-Hoeksema S. Emotion dysregulation and adolescent psychopathology: a prospective study. Behav Res Ther. (2011) 49:544–54. 10.1016/j.brat.2011.06.003 21718967 PMC3153591

[22] Twenge JM Nolen-Hoeksema S. Age, gender, race, socioeconomic status, and birth cohort difference on the children's depression inventory: a meta-analysis. J Abnorm Psychol. (2002) 111:578–88. 10.1037/0021-843X.111.4.578 12428771

[23] Costello EJ Copeland W Angold A. Trends in psychopathology across the adolescent years: what changes when children become adolescents, and when adolescents become adults? J Child Psychol Psychiatry. (2011) 52:1015–25. 10.1111/j.1469-7610.2011.02446.x 21815892 PMC3204367

[24] King KM Chassin L. Adolescent stressors, psychopathology, and young adult substance dependence: A prospective study. J Stud Alcohol Drugs. (2008) 69:629–38. 10.15288/jsad.2008.69.629 18781237 PMC2575393

[25] Leebens PK Williamson ED. Developmental psychopathology: risk and resilience in the transition to young adulthood. Child Adolescent Psychiatr Clin. (2017) 26:143–56. 10.1016/j.chc.2016.12.001 28314447

[26] Howard LM Piot P Stein A. No health without perinatal mental health. Lancet. (2014) 384:1723–4. 10.1016/S0140-6736(14)62040-7 25455235

[27] Glynn LM Howland MA Fox M. Maternal programming: application of a developmental psychopathology perspective. Dev Psychopathol. (2018) 30:905–19. 10.1017/S0954579418000524 30068423 PMC6274636

[28] Walker EF. Adolescent neurodevelopment and psychopathology. Curr Dir Psychol Sci. (2002) 11:24–8. 10.1111/1467-8721.00161

[29] Fitzpatrick C Barnett T Pagani LS. Early exposure to media violence and later child adjustment. J Dev Behav Pediatr. (2012) 33:291–7. 10.1097/DBP.0b013e31824eaab3 22481072

[30] Gunnar MR Wewerka S Frenn K Long JD Griggs C. Developmental changes in hypothalamus–pituitary–adrenal activity over the transition to adolescence: normative changes and associations with puberty. Dev Psychopathol. (2009) 21:69–85. 10.1017/S0954579409000054 19144223 PMC3933029

[31] Lewis DA Sesack SR Levey AI Rosenberg DR. Dopamine axons in primate prefrontal cortex: specificity of distribution, synaptic targets, and development. Adv Pharmacol. (1997) 42:703–6. 10.1016/S1054-3589(08)60845-5 9327996

[32] Wahlstrom D Collins P White T Luciana M. Developmental changes in dopamine neurotransmission in adolescence: behavioral implications and issues in assessment. Brain Cogn. (2010) 72:146–59. 10.1016/j.bandc.2009.10.013 19944514 PMC2815132

[33] Guerry JD Hastings PD. In search of HPA axis dysregulation in child and adolescent depression. Clin Chil Fam Psychol Rev. (2011) 14:135–60. 10.1007/s10567-011-0084-5 21290178 PMC3095794

[34] Brown WJ Wilkerson AK Boyd SJ Dewey D Mesa F Bunnell BE. A review of sleep disturbance in children and adolescents with anxiety. J Sleep Res. (2018) 27:e12635. 10.1111/jsr.12635 29193443

[35] Aledavood T Torous J Hoyos AMT Naslund JA Onnela Keshavan M. Smartphone-based tracking of sleep in depression, anxiety, and psychotic disorders. Curr Psychiatry Rep. (2019) 21:49. 10.1007/s11920-019-1043-y 31161412 PMC6546650

[36] McRae K Gross JJ Weber J Robertson ER Sokol-Hessner P Ray RD Ochsner KN. The development of emotion regulation: an fMRI study of cognitive reappraisal in children, adolescents and young adults. Soc Cogn Affect Neurosci. (2012) 7:11–22. 10.1093/scan/nsr093 22228751 PMC3252634

[37] Lemola S Perkinson-Gloor N Brand S Dewald-Kaufmann JF Grob A. Adolescents' electronic media use at night, sleep disturbance, and depressive symptoms in the smartphone age. J Youth Adolesc. (2015) 44:405–18. 10.1007/s10964-014-0176-x 25204836

[38] Wall K Vanwoerden S Penner F Patriquin M Alfano CA Sharp C. Adolescent sleep disturbance, emotion regulation and borderline features in an inpatient setting. J Clin Child Adolesc Psychol. (2020) 1–15. 10.1080/15374416.2020.1772081 32603239

[39] Faurholt-Jepsen M Frost M Vinberg M Christensen EM Bardram JE Kessing LV. Smartphone data as objective measures of bipolar disorder symptoms. Psychiatry Res. (2014) 217:124–7. 10.1016/j.psychres.2014.03.009 24679993

[40] Staples P Torous J Barnett I Carlson K Sandoval L Keshavan M. A comparison of passive and active estimates of sleep in a cohort with schizophrenia. NPJ Schizophr. (2017) 3:1–6. 10.1038/s41537-017-0038-0 29038553 PMC5643440

[41] Walch OJ Cochran A Forger DB. A global quantification of “normal” sleep schedules using smartphone data. Sci Adv. (2016) 2:e1501705. 10.1126/sciadv.1501705 27386531 PMC4928979

[42] Rohani DA Faurholt-Jepsen M Kessing LV Bardram JE. Correlations between objective behavioral features collected from mobile and wearable devices and depressive mood symptoms in patients with affective disorders: systematic review. JMIR mHealth uHealth. (2018) 6:e165. 10.2196/mhealth.9691 30104184 PMC6111148

[43] Wisniewski H Henson P Torous J. Using a smartphone app to identify clinically relevant behavior trends via symptom report, cognition scores, and exercise levels: a case series. Front Psychiatry. (2019) 10:652. 10.3389/fpsyt.2019.00652 31607960 PMC6767851

[44] Twenge JM Joiner TE Rogers ML Martin GN. Increases in depressive symptoms, suicide-related outcomes, and suicide rates among U.S. adolescents after 2010 and links to increased new media screen time. Clin Psychol Sci. (2018) 6:3–17. 10.1177/2167702617723376

[45] Elhai JD Dvorak RD Levine JC Hall BJ. Problematic smartphone use: a conceptual overview and systematic review of relations with anxiety and depression psychopathology. J Affect Disord. (2017) 207:251–9. 10.1016/j.jad.2016.08.030 27736736

[46] Chun J. Effects of psychological problems, emotional dysregulation, and self-esteem on problematic Internet use among Korean adolescents. Chil Youth Serv Rev. (2016) 68:187–92. 10.1016/j.childyouth.2016.07.005

[47] Marino C Gini G Angelini F Vieno A Spada MM. Social norms and e-motions in problematic social media use among adolescents. Addict Behav Rep. (2020) 11:100250. 10.1016/j.abrep.2020.100250 32467839 PMC7244919

[48] Yu JJ Kim H Hay I. Understanding adolescents' problematic Internet use from a social/cognitive and addiction research framework. Comput Hum Behav. (2013) 29:2682–9. 10.1016/j.chb.2013.06.045

[49] McMahon EM Corcoran P O'Regan G Keeley H Cannon M Carli V. Physical activity in European adolescents and associations with anxiety, depression and well-being. Eur Child Adolesc Psychiatry. (2017) 26:111–22. 10.1007/s00787-016-0875-9 27277894

[50] Norris R Carroll D Cochrane R. The effects of physical activity and exercise training on psychological stress and well-being in an adolescent population. J Psychosom Res. (1992) 36:55–65. 10.1016/0022-3999(92)90114-H 1538350

[51] Adrian M. , Zeman J, Veits G. Methodological implications of the affect revolution: a 35-year review of emotion regulation assessment in children. J Exp Child Psychol. (2011) 110:171–97. 10.1016/j.jecp.2011.03.009 21514596

[52] Tamminen KA Gaudreau P McEwen CE Crocker PR. Interpersonal emotion regulation among adolescent athletes: a Bayesian multilevel model predicting sport enjoyment and commitment. J Sport Exerc Psychol. (2016) 38:541–55. 10.1123/jsep.2015-0189 27383379

[53] Dishion TJ Tipsord JM. Peer contagion in child and adolescent social and emotional development. Annu Rev Psychol. (2011) 62:189–214. 10.1146/annurev.psych.093008.100412 19575606 PMC3523739

[54] Byrnes HF Miller BA Morrison CN Wiebe DJ Remer LG Wiehe. Brief report: using global positioning system (GPS) enabled cell phones to examine adolescent travel patterns and time in proximity to alcohol outlets. J Adolesc. (2016) 50:65–8. 10.1016/j.adolescence.2016.05.001 27214713

[55] Hochberg Z Konner M. Emerging adulthood, a pre-adult life-history stage. Front Endocrinol. (2019) 10:918. 10.3389/fendo.2019.00918 31993019 PMC6970937

[56] Schulenberg JE Zarrett NR. Mental health during emerging adulthood: continuity and discontinuity in courses, causes, and functions. In: Arnett JJ Tanner JL editors. Emerging Adults in America: Coming of Age in the 21st Century. Washington, DC: American Psychological Association (2006). p. 135–72. 10.1037/11381-006

[57] Caplan SE. Relations among loneliness, social anxiety, and problematic internet use. CyberPsychol Behav. (2007) 10:234–42. 10.1089/cpb.2006.9963 17474841

[58] Meers JM Bower JL Alfano CA. Poor sleep and emotion dysregulation mediate the association between depressive and premenstrual symptoms in young adult women. Arch Women's Ment Health. (2019) 23:351–9. 10.1007/s00737-019-00984-2 31214782

[59] Semplonius T Willoughby T. Psychosocial adjustment throughout university: a longitudinal investigation of the roles of sleep quality and emotion dysregulation. J Youth Adolesc. (2018) 47:1267–78. 10.1007/s10964-018-0826-5 29476457

[60] Casale S Caplan SE Fioravanti G. Positive metacognitions about Internet use: the mediating role in the relationship between emotional dysregulation and problematic use. Addictive Behav. (2016) 59:84–8. 10.1016/j.addbeh.2016.03.014 27077964

[61] Hormes JM Kearns B Timko CA. Craving facebook? Behavioral addiction to online social networking and its association with emotion regulation deficits. Addiction. (2014) 109:2079–88. 10.1111/add.12713 25170590

[62] Saeb S Zhang M Karr CJ Schueller SM Corden ME Kording KP. Mobile phone sensor correlates of depressive symptom severity in daily-life behavior: an exploratory study. JMIR. (2015) 17:e175. 10.2196/jmir.4273 26180009 PMC4526997

[63] Saeb S Lattie EG Kording KP Mohr DC. Mobile phone detection of semantic location and its relationship to depression and anxiety. JMIR mHealth uHealth. (2017) 5:e112. 10.2196/mhealth.7297 28798010 PMC5571235

[64] Raugh IM James SH Gonzalez CM Chapman HC Cohen AS Kirkpatrick B. Geolocation as a digital phenotyping measure of negative symptoms and functional outcome. Schizophr Bull. (2020) 46:1596–607. 10.1093/schbul/sbaa121 32851401 PMC7751192

[65] Niven K Garcia D Van der Löwe I Holman D Mansell W. Becoming popular: interpersonal emotion regulation predicts relationship formation in real life social networks. Front Psychol. (2015) 6:1452. 10.3389/fpsyg.2015.01452 26483718 PMC4586352

[66] Gross JJ John OP. Individual differences in two emotion regulation processes: implications for affect, relationships, and well-being. J Pers Soc Psychol. (2003) 85:348–62. 10.1037/0022-3514.85.2.348 12916575

[67] Marroquín B Tennen H Stanton AL. Coping, emotion regulation, and well-being: intrapersonal and interpersonal processes. In: Robinson M Eid M editors. The Happy Mind: Cognitive Contributions to Well-Being. Cham: Springer (2017). p. 253–74. 10.1007/978-3-319-58763-9_14

[68] Hoffner CA Lee S. Mobile phone use, emotion regulation, and well-being. Cyberpsychol Behav Soc Netw. (2015) 18:411–6. 10.1089/cyber.2014.0487 26167841

[69] Oulasvirta A Rattenbury T Ma L Raita E. Habits make smartphone use more pervasive. Pers Ubiquitous Comput. (2012) 16:105–14. 10.1007/s00779-011-0412-2

[70] Billieux J Maurage P Lopez-Fernandez O Kuss DJ Griffiths MD. Can disordered mobile phone use be considered a behavioral addiction? An update on current evidence and a comprehensive model for future research. Curr Addict Rep. (2015) 2:156–62. 10.1007/s40429-015-0054-y

[71] Kim JH Seo M David P. Alleviating depression only to become problematic mobile phone users: can face-to-face communication be the antidote? Comput Hum Beha.v. (2015) 51:440–7. 10.1016/j.chb.2015.05.030

[72] Beckes L Coan JA. Social baseline theory: the role of social proximity in emotion and economy of action. Soc Personal Psychol Compass. (2011) 5:976–88. 10.1111/j.1751-9004.2011.00400.x

[73] Woods SM Melville JL Guo Y Fan MY Gavin A. Psychosocial stress during pregnancy. Am J Obstet Gynecol. (2010) 202:61.e1–61.e7. 10.1016/j.ajog.2009.07.041 19766975 PMC2811236

[74] Marcus SM Flynn HA Blow FC Barry KL. Depressive symptoms among pregnant women screened in obstetrics settings. J Women's Health. (2003) 12:373–80. 10.1089/154099903765448880 12804344

[75] Newham JJ Martin CR. Measuring fluctuations in maternal well-being and mood across pregnancy. J Reprod Infant Psychol. (2013) 31:531–40. 10.1080/02646838.2013.834040

[76] O'Hara MW Wisner KL. Perinatal mental illness: definition, description and aetiology. Best Pract Res Clin Obstet Gynaecol. (2014) 28:3–12. 10.1016/j.bpobgyn.2013.09.002 24140480 PMC7077785

[77] Ostlund BD Vlisides-Henry RD Crowell SE Raby KL Terrell S Brown MA. Intergenerational transmission of emotion dysregulation: Part II. developmental origins of newborn neurobehavior. Dev Psychopathol. (2019) 31:831–45. 10.1017/S0954579419000440 31057128 PMC6790984

[78] Doyle C Cicchetti D. Future directions in prenatal stress research: challenges and opportunities related to advancing our understanding of prenatal developmental origins of risk for psychopathology. Dev Psychopathol. (2018) 30:721–4. 10.1017/S095457941800069X 30068432

[79] Facco FL Kramer J Ho KH Zee PC Grobman WA. Sleep disturbances in pregnancy. Obstet Gynecol. (2010) 115:77–83. 10.1097/AOG.0b013e3181c4f8ec 20027038

[80] Okun ML Hanusa BH Hall M Wisner KL. Sleep complaints in late pregnancy and the recurrence of postpartum depression. Behav Sleep Med. (2009) 7:106–17. 10.1080/15402000902762394 19330583 PMC2909658

[81] Posmontier B. Sleep quality in women with and without postpartum depression. J Obstet Gynecol Neonatal Nurs. (2008) 37:722–37. 10.1111/j.1552-6909.2008.00298.x 19012723 PMC2597421

[82] Ross LE Murray BJ Steiner M. Sleep and perinatal mood disorders: a critical review. J Psychiatry Neurosci. (2005) 30:247–56. 16049568 PMC1160560

[83] Faherty LJ Hantsoo L Appleby D Sammel MD Bennett IM Wiebe. Movement patterns in women at risk for perinatal depression: use of a mood-monitoring mobile application in pregnancy. J Am Med Inform Assoc. (2017) 24:746–53. 10.1093/jamia/ocx005 28339686 PMC6580935

[84] Stapleton LRT Schetter CD Westling E Rini C Glynn LM. Perceived partner support in pregnancy predicts lower maternal and infant distress. J Fam Psychol. (2012) 26:453–63. 10.1037/a0028332 22662772 PMC3992993

[85] Schatten HT Allen KJD Armey MF. Assessment of emotion dysregulation using ecological momentary assessment. In: Beauchaine TP Crowell SE editors. The Oxford Handbook of Emotion Dysregulation. New York, NY: Oxford University Press (2020). p. 411–26. 10.1093/oxfordhb/9780190689285.013.29

[86] Kahn M Sheppes G Sadeh A. Sleep and emotions: bidirectional links and underlying mechanisms. Int J Psychophysiol. (2013) 89:218–28. 10.1016/j.ijpsycho.2013.05.010 23711996

[87] Kleiman EM Glenn CR Liu RT. Real-time monitoring of suicide risk among adolescents: potential barriers, possible solutions, and future directions. J Clin Child Adolesc Psychol. (2019) 48:934–46. 10.1080/15374416.2019.1666400 31560584 PMC6864279

[88] Tan X Shiyko MP Li R Li Y Dierker L. A time-varying effect model for intensive longitudinal data. Psychol Methods. (2012) 17:61–77. 10.1037/a0025814 22103434 PMC3288551

[89] Mishra T Wang M Metwally AA Bogu GK Brooks AW Bahmani A. Early detection of COVID-19 using a smartwatch. medRxiv [Preprint]. (2020), 1–31. 10.1101/2020.07.06.20147512 PMC9020268 33208926

[90] Allen NB Nelson BW Brent D Auerbach RP. Short-term prediction of suicidal thoughts and behaviors in adolescents: can recent developments in technology and computational science provide a breakthrough? J Affect Disord. (2019) 250:163–9. 10.1016/j.jad.2019.03.044 30856493 PMC6481940

[91] Vlisides-Henry RD Crowell SE Kaufman EA Lin B. Social processes and dyadic designs. In: Wright AGC Hallquist MN editors. The Cambridge Handbook of Research Methods in Clinical Psychology. Cambridge: Cambridge University Press (2020). p. 337–49. 10.1017/9781316995808.032

[92] Martinez-Martin N Insel TR Dagum P Greely HT Cho MK. Data mining for health: staking out the ethical territory of digital phenotyping. NPJ Digit. (2018) 1:1–5. 10.1038/s41746-018-0075-8 31211249 PMC6550156

[93] Sequeira L Battaglia M Perrotta S Merikangas K Strauss J. Digital phenotyping with mobile and wearable devices: advanced symptom measurement in child and adolescent depression. J Am Acad Child Adolesc Psychol. (2019) 58:841–5. 10.1016/j.jaac.2019.04.011 31445619

[94] Caplan SE. Problematic Internet use and psychosocial well-being: development of a theory-based cognitive–behavioral measurement instrument. Comput Hum Behav. (2002) 18:553–75. 10.1016/S0747-5632(02)00004-3

